# O-GlcNAcylation in ischemic diseases

**DOI:** 10.3389/fphar.2024.1377235

**Published:** 2024-05-09

**Authors:** Rui-Rui Shi, Tian-Qi He, Meng-Si Lin, Jian Xu, Jin-Hua Gu, Hui Xu

**Affiliations:** ^1^ Nantong Institute of Genetics and Reproductive Medicine, Affiliated Maternity and Child Healthcare Hospital of Nantong University, Nantong, China; ^2^ Department of Pharmacy, Affiliated Maternity and Child Healthcare Hospital of Nantong University, Nantong, China; ^3^ Prenatal Screening and Diagnosis Center, Affiliated Maternity and Child Healthcare Hospital of Nantong University, Nantong, China

**Keywords:** O-GlcNAcylation, ischemic diseases, chronic metabolic diseases, post-translational modification, diabetes

## Abstract

Protein glycosylation is an extensively studied field, with the most studied forms being oxygen or nitrogen-linked N-acetylglucosamine (O-GlcNAc or N-GlcNAc) glycosylation. Particular residues on proteins are targeted by O-GlcNAcylation, which is among the most intricate post-translational modifications. Significantly contributing to an organism’s proteome, it influences numerous factors affecting protein stability, function, and subcellular localization. It also modifies the cellular function of target proteins that have crucial responsibilities in controlling pathways related to the central nervous system, cardiovascular homeostasis, and other organ functions. Under conditions of acute stress, changes in the levels of O-GlcNAcylation of these proteins may have a defensive function. Nevertheless, deviant O-GlcNAcylation nullifies this safeguard and stimulates the advancement of several ailments, the prognosis of which relies on the cellular milieu. Hence, this review provides a concise overview of the function and comprehension of O-GlcNAcylation in ischemia diseases, aiming to facilitate the discovery of new therapeutic targets for efficient treatment, particularly in patients with diabetes.

## 1 Introduction

Ischemic diseases, including acute myocardial infarction (MI) and stroke have a rapid onset, aggressive course, and poor prognosis. These diseases have become a focus of attention in current medical research because of an increasing level of public health awareness. Recently, research on the molecular processes related to injury in ischemic diseases has deepened and the modification of oxygen-linked N-acetylglucosamine (O-Linked β-N-acetylglucosamine, O-GlcNAc) has gained increased attention as a special protein modification widely present in the cell’s nucleus and cytoplasm (Jin et al., 2021a). Increasing the degree of intracellular O-GlcNAc modification can enhance cellular stress tolerance and plays a role in resisting oxidative stress injury, promoting cellular autophagy, maintaining the balance of mitochondrial energy metabolism, and reducing apoptosis in ischemic disease injury (Watson et al., 2010; Lima et al., 2012; Xu et al., 2019; Akinbiyi et al., 2021; Xu et al., 2022).

An instance of post-translational modification, known as O-GlcNAcylation, occurs when a solitary N-acetylglucosamine (GlcNAc) molecule is attached to a target protein at a serine or threonine residue. This alteration is crucial to numerous biological processes, including stress responses, signaling, and gene expression. The function of O-GlcNAcylation in ischemic diseases, including heart disease and stroke, remains unknown.

O-GlcNAcylation regulates numerous cellular processes, including glucose metabolism, inflammation, and oxidative stress, which are all disrupted in ischemic diseases. Ischemic injury induces changes in O-GlcNAcylation levels, and manipulating this modification may confer protective effects in animal models of ischemic diseases. While there are instances where significant increases in O-GlcNAcylation may promote the resolution of diseases, an expanding body of evidence indicates that prolonged excessive increases in O-GlcNAcylation worsens the development of numerous chronic conditions (Very et al., 2018; Herrero-Beaumont and Largo, 2020; Spaner, 2021; Silva-Aguiar et al., 2022; Fan et al., 2023; Wang et al., 2023). Advancements in our understanding of protein O-GlcNAcylation in ischemic diseases have yielded significant knowledge regarding the molecular processes that govern these conditions and have also identified prospective novel therapeutic targets.

Our review is exclusively focused on the role of O-GlcNAcylation in ischemic diseases, offering detailed insights into the specific molecular and cellular mechanisms underlying these conditions. By exploring the potential translational and clinical implications of O-GlcNAcylation within the context of ischemic diseases, we aim to bridge the gap between basic research and clinical application. This targeted approach distinguishes our work from broader reviews on O-GlcNAcylation. Through an examination of how proteins undergo O-GlcNAc modification and the significance of O-GlcNAc in ischemia-related disorders, our review aims to provide innovative ideas for basic research and propose novel preventive and therapeutic strategies for the clinical management of ischemic injuries.

## 2 Mechanisms of protein O-GlcNAcylation modification

O-GlcNAcylation is a post-translational modification that occurs on serine and threonine residues of proteins located in the mitochondria, cytoplasm, and nucleus (Torres and Hart, 1984; Li et al., 2023). It is a nutrient-driven, dynamic, and reversible process that is essential for regulation of cellular function (Wang et al., 2008). O-GlcNAcylation serves as a nutrient sensor and is dependent on the concentration of uracil-N-acetylglucosamine diphosphate (UDP-GlcNAc). Changes in extracellular nutrition can result in slight changes in UDP-GlcNAc concentration, which in turn significantly alters the level of O-GlcNAcylation. This allows the cell to appropriately respond to changes in external conditions and stress (Liu et al., 2021b). Autophagy, epigenetic signaling, Ca^2+^ management, protein synthesis, quality control and turnover of synthetic proteins, transcription and translation, metabolism, and mitochondrial function are among the numerous cellular processes regulated by O-GlcNAcylation (Wright et al., 2017).

The process of O-GlcNAcylation, which involves the addition of a sugar molecule to proteins, occurs specifically within the cytoplasm, mitochondria and nucleus of cells, rather than on the outer surface of the cell. The regulation of O-GlcNAc levels is highly controlled by the availability of glucose, as it is a process driven by nutrients that is particularly reliant on changes in glucose availability. A minute proportion of the glucose that enters the cell, specifically around 2%–5%, is directed into the hexosamine biosynthesis pathway (HBP).

Glucose undergoes a series of metabolic steps beginning with the conversion of glucose to fructose 6-phosphate via the action of glucokinase; a translocase converts fructose 6-phosphate to glucosamine 6-phosphate; and glutamine fructose-6-phosphate aminotransferase (GFAT), a rate-limiting enzyme catalysing the conversion to glucosamine 6-phosphate; and finally, this pathway culminates in the production of protein O-GlcNAcylation substrates UDP-GlcNAc and facilitates other glycosylation forms.

The biosynthesis of UDP-GlcNAc, the donor substrate for O-GlcNAc transferase (OGT), is intricately linked to nutrient availability and the flux through primary metabolic pathways due to its connection with the HBP, encompassing glucose, nitrogen, nucleotide, and fatty acid metabolism. This positioning allows UDP-GlcNAc, and subsequently O-GlcNAcylation, to act as pivotal connectors among these pathways, highlighting O-GlcNAcylation’s essential role as a nutrient sensor. A portion of UDP-GlcNAc attaches individual GlcNAc to protein serine or threonine residues in a β-O-linked manner and forms an O-glycosylation modification (O-GlcNAcylation modification) by the action of OGT. Simultaneously, O-GlcNAcase (OGA) can hydrolyze GlcNAc from protein hydroxyl groups, with *in vivo* O-GlcNAc levels maintained in a delicate and dynamic equilibrium by the action of the circulating enzyme OGT/OGA. In addition, OGT activity is vulnerable to variations in UDP-GlcNAc. Consequently, fluctuations in UDP-GlcNAc availability also impact O-GlcNAc levels.

More specific regulators were discovered recently and these regulators have provided novel ideas to study O-GlcNAc modification. With the growing recognition of the crucial role that O-GlcNAcylation plays in the regulatory network of cellular biological processes, its significance as a bridge between extracellular stimuli and cellular stress responses, and as a regulator of cellular signal transduction, provides an increasingly novel direction for research into the treatment of ischemic injury.

### 2.1 GFAT is the rate-limiting enzyme in hexosamine biosynthesis

GFAT is the primary rate-limiting enzyme in the HBP. GFAT exhibits distinct expression and functionality in various tissues and organs. Specifically, GFAT1 is predominant in the pancreas, placenta, testis, and skeletal muscle, while GFAT2 predominates in the heart and central nervous system. GFAT is controlled at different levels, including allosteric regulation by metabolites, post-translational modifications, and regulation of mRNA and protein expression. This regulation ensures the coordination of *de novo* hexosamine biosynthesis with other metabolic pathways in response to nutrient availability, as well as the presence of appropriate environmental and cellular signals. UDP-GlcNAc binds to GFAT1 to negatively regulate its activity. Mutations in GFAT1, including G451E, expressed in *Caenorhabditis elegans* and mouse neuroblastoma cells, can prevent feedback inhibition by UDP-GlcNAc (Ruegenberg et al., 2020). Phosphorylation of GFAT1 at Ser205, Ser235, and Ser243 also regulates its activity. PKA mediates phosphorylation at Ser205, which is conserved in both GFAT1 and GFAT2 (Ser202 in the latter) (Hu et al., 2004). PKA-mediated phosphorylation at Ser205 stabilizes the glutamine amidotransferase and isomerase domains of GFAT1, while preventing UDP-GlcNAc feedback inhibition, thereby enhancing enzyme activity (Ruegenberg et al., 2021).

The GFAT antagonists L-6-diazo-5-oxonorleucine (DON) and O-diazoacetyl-L-serine (Azaserine), commonly used in current studies, irreversibly inhibit GFAT, leading to a decrease in cellular allocation of glucose to the HBP pathway.

**FIGURE 1:**
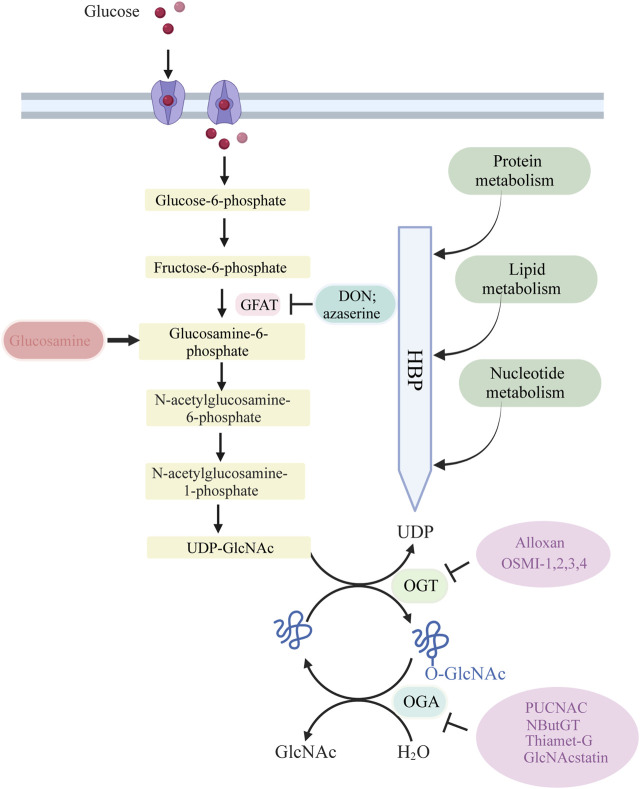
Glucose is taken up from the extracellular environment by the glucose transporter (GLUT) protein. While most glucose is used for glycolysis and glycogen synthesis, approximately 2%–5% is delivered to the HBP. GFAT catalyzes the rate-limiting step of HBP, converting fructose 6-phosphate to glucosamine 6-phosphate. Subsequent acetylation and uridylation of GlcN-6P produce the donor substrate for protein O-GlcNAcylation, UDP-GlcNA. OGT and OGA catalyze the addition and removal of O-GlcNAc to complete the O-GlcNAcylation of target proteins.

Cellular O-GlcNAcylation is believed to increase with the availability of nutrients essential for UDP-GlcNAc biosynthesis via the HBP, including glucose, glutamine, and acetyl coenzyme (Yang and Qian, 2017). Almost all metabolic pathways contribute to UDP-GlcNAc production, including fatty acids, nitrogen, and approximately 2%–5% of glucose. An ample supply of energy leads to elevated UDP-GlcNAc levels and subsequent protein O-GlcNAcylation, while a scarcity of these nutrients results in reduced UDP-GlcNAc and O-GlcNAc levels (Hart et al., 2007; Hart et al., 2011). To-date, numerous research groups have observed this phenomenon, with high glucose levels enhancing cellular O-GlcNAc glycosylation across various tissues in response to the regulation of glucose concentration (Shao and Bayraktutan, 2014). Studies have also shown that even low concentrations of glucosamine (GlcN), a metabolite that by-passes the rate-limiting step of HBP by converting fructose-6-phosphate to glucosamine-6-phosphate via GFAT (Figure 1), can significantly augment O-GlcNAcylation. This further supports the notion that HBP flux plays a critical role in determining rates of cellular O-GlcNAcylation.

One potential mechanism involves the upregulation of OGT gene expression, which can elevate cellular O-GlcNAcylation levels even in the presence of reduced intracellular UDP-GlcNAc availability. This phenomenon cannot be solely attributed to changes in HBP flux (Cheung and Hart, 2008). Thus, the regulation of O-GlcNAc signaling extends beyond controlling UDP-GlcNAc abundance via HBP flux. It also encompasses the modulation of OGT, OGA, and their associated target proteins and substrates.

### 2.2 O-GlcNAc cyclase

O-GlcNAc undergoes dynamic addition and removal through the action of two cycling enzymes, OGT and OGA, resembling a binary “on” or “off” switch. This notion finds support in the fact that only two enzymes, OGT and OGA, are responsible for the addition and removal of O-GlcNAc to/from proteins (Cook et al., 2023). Furthermore, in certain cellular contexts, the regulation of overall O-GlcNAc levels occurs by modulating these two enzymes. Lu et al. (2023) reconstructed the full-length human OGT-OGA complex and determined its cryo-electron microscopy structure, which revealed the mutual enzymatic inhibition of OGT and OGA *in vitro*. This direct mutual inhibition of the enzyme pair at the protein level helps to maintain steady-state levels of O-GlcNAcylation.

#### 2.2.1 O-GlcNAc transferase (OGT)

OGT is a highly conserved glycosyltransferase found in the nucleus and cytoplasm, comprising two significant structural domains. The N-terminal contains multiple tetrapeptide (TPR) repeats, while the C-terminal binds to UDP-GlcNAc and possesses glycosyltransferase activity. OGT catalyzes a sequential process, initially binding to UDP-GlcNAc and subsequently to the substrate (Lazarus et al., 2011; Banerjee et al., 2013). OGT has three major isoforms: Nucleocytoplasmic OGT, which is the full-length variant with 11–12 TPRs depending on the species; mitochondrial OGT, which starts with a mitochondrial targeting sequence, a transmembrane region, and the last nine TPRs found in Nucleocytoplasmic OGT; and soluble OGT, containing only three TPRs (Hanover et al., 2003). Most studies indicate the presence of only Nucleocytoplasmic OGT in total brain lysates, with the brain displaying the highest O-GlcNAc expression (März et al., 2006). OGT is tightly regulated, selectively adding O-GlcNAc to specific sites in a temporal and spatial manner. *In vitro*, OGT exhibits sequence specificity, modifying one or a few sites when mixed with UDP-GlcNAc and a peptide containing multiple possible O-GlcNAc sites. Different substrate peptides from various proteins are typically modified with varying efficiencies (Kreppel and Hart, 1999). OGT is a crucial cellular trophic sensor; the modification of a specific protein is also contingent on the protein to which OGT binds. OGT functions as a holoenzyme, with its interacting proteins guiding it to its substrates (Yang et al., 2002; Cheung et al., 2008; Housley et al., 2009). Furthermore, OGT undergoes multi-phosphorylation and can self-activate through O-GlcNAc modification (Hart, 1997; Song et al., 2008). Although OGT can be affected by phosphorylation and glycosylation, specific pharmacological approaches to increase OGT activity do not exist. While OGT inhibitors, including Alloxan, 4-methoxyphenyl 6- acetyl-2-oxobenzo [d]oxazole-3(2H)-carboxylate (BZX), and Benzyl-2-acetamido-2-deoxy-α-d-galactopyranoside (BADGP) have been used in experimental studies and they are not recommended for clinical use due to numerous off-target effects and toxicity (Lenzen and Panten, 1988; Hennebicq-Reig et al., 1998; Ostrowski and van Aalten, 2013). Alloxan is an *in vitro* inhibitor of OGT and gains entry into pancreatic beta cells through the glucose transporter GLUT2, although it is not exclusively targeted at OGT, as it also inhibits OGA (Lee et al., 2006). The absence of selective OGT inhibitors has impeded our understanding of O-GlcNAc biology and the creation of clinically viable drugs. Nevertheless, recent developments have led to more promising OGT inhibitors, including OSMI 1 [(R)-N-(furan-2-ylmethyl)-2-(2-methoxyphenyl)-2-(2-oxo-1,2-dihydroquinoline-6-sulfonamido)- N-(thiophen-2-ylmethyl)acetamide] and OSMI 2-4, which offer improved specificity and potential as pharmacological agents (Ortiz-Meoz et al., 2015; Martin et al., 2018).

#### 2.2.2 O-GlcNAcase (OGA)

To-date, OGA has been identified in every tissue examined. Its expression is greatest in the brain, where it is found in both the nucleus and cytoplasm (Gao et al., 2001). Despite being highly conserved, it does comprise a region that is intrinsically unfolded and serves as an intermediate, demonstrating great variability (Heckel et al., 1998; Butkinaree et al., 2008). OGA, similar to OGT (the enzyme responsible for adding O-GlcNAc to proteins), is encoded by a single gene and comprises two primary domains. The C-terminal domain includes the glycosidase structural component, while the N-terminus contains a histone acetyltransferase structural domain (Toleman et al., 2004; Toleman et al., 2006). Structural data have recently revealed that the O-GlcNAcase possesses an active site with significant structural similarity to the human lysosomal hexosaminidases HexA/HexB (Dorfmueller et al., 2006).

Recently, pharmacological inhibitors of OGA were developed and extensively employed to enhance O-GlcNAcylation in cultured cells and animal models (Yuzwa et al., 2008; Yuzwa et al., 2012). A series of studies identified several OGA inhibitors; O-(2-acetamido-2-deoxy-D-gluco-pyranosylidene) (PUGNAc) was initially characterized as another inhibitor of b-N-acetylhexosidase and later identified as an OGA inhibitor, which also significantly inhibits other hexosaminidases (Horsch et al., 1991; Dong and Hart, 1994). Subsequent *in vivo* and *in vitro* experiments have revealed that 1,2-dideoxy-2′-propyl-α-d-glucopyranoso-[2,1-D]-Δ2′-thiazoline (NButGT) is a more efficient enhancer of O-GlcNAcylation (Ji et al., 2012; Mehdy et al., 2012; Shan et al., 2012). 5H-Pyrano [3,2-d] thiazole-6,7-diol,2-(ethylamino)-3a,6,7,7a-tetrahydro-5-(hydroxymethyl) (3aR,5R,6S,7R,7aR) (Thiamet G), a compound that is highly selective for human OGA, and the recently developed GlcNAcstatin (Horsch et al., 1991; Dorfmueller et al., 2006; Dorfmueller et al., 2009; Yu et al., 2012). Thiamet G exhibits remarkable stability in solution and can penetrate the blood-brain barrier, rendering it highly appropriate for application in both cell culture and intact animal models. GlcNAcstatins, are a novel family of potent human OGA inhibitors with significant similarity to the molecular architecture of PUGNAc (Dorfmueller et al., 2006).

The pharmacological tools mentioned above for regulating O-GlcNAc levels are summarized in Table 1.

**TABLE 1:** Pharmacological tools to modulate O-GlcNAc levels.

Modulate O-GlcNAc levels	Pharmacological tools	Characteristics	Commercial availability
GFAT inhibitor	DON	Decrease HBP flux by inhibiting GFAT. However, it has low selectivity for GFAT as it can inhibit other pathways that utilize glutamine	Multiple sources
	Azaserine	Decrease HBP flux by inhibiting GFAT. However, it has low selectivity for GFAT as it can inhibit other pathways that utilize glutamine	Multiple sources
OGT inhibitor	Alloxan	A weak OGT inhibitor with numerous off target effects. Requires millimolar concentrations to lower cellular O-GlcNAc levels	Multiple sources
	BZX	BZX competes with the binding of sugar nucleotides. It is a suicide inhibitor that cross-links the active site of OGT (Lys842 and Cys917) through a double-displacement mechanism	No
	BADGP	BADGP decreases O-GlcNAc levels in millimolar range. However, it lacks specificity for OGT and it may inhibit other O-Glycosyltransferases	Sigma
	OSMI 1	Reduces O-GlcNAc levels in cells. Shown to be specific for OGT compared with other glycosyltranserases	Sigma
	OSMI 2–4	On the basis of OSMI-1 scaffold, but with greater potency, these compounds selectively inhibit OGT and the active forms of OSMI 3 and OSMI 4 have low nanomolar binding affinities, a milestone in the development of glycosyltransferase inhibitors	ProbeChem
OGA inhibitor	PUGNAc	Widely used cell permeable OGA inhibitor, but also inhibits hexosaminidase A and B inhibitor	Multiple sources
	NButGT	OGA inhibitor based on NAG-thiazoline scaffold. Less potent that NAG-thiazoline but even more selective than other hexosaminidases	No
	Thiamet G	Derivative of NButGT, with greater stability, water soluble, and orally available	Multiple sources
	GlcNAcstatins	GlcNAcstatin is a glucoimidazole with noticeable similarity to the molecular architecture of PUGNAc, but bearing a larger iso-butanamido group on N8 and a phenethyl group on C2	Abmole Bioscience, Inc

In addition to these pharmacological tools, specific antibodies targeting O-GlcNAcylation have been developed. Although only a limited number of antibodies have been identified to target specific site (Comer et al., 2001). Among them, CTD110.6 and RL2 are widely utilized for various applications, including immunoblotting, immunofluorescence, and immunohistochemistry. Each of these chemistries offers the opportunity to manipulate and detect O-GlcNAcylation in cellular and animal disease models.

## 3 Role of protein O-GlcNAcylation in different ischemic diseases

### 3.1 Role of O-GlcNAcylation in ischemic stroke

O-GlcNAcylation occurs in the context of multiple proteins targeted to the nucleus, cytoplasm, and mitochondria. O-GlcNAc is prominently present in the nervous system, particularly in the brain, which exhibits a high concentration of O-GlcNAc. The brain exhibits relatively high levels of expression for OGT and OGA, with OGT being particularly abundant in neural synapses (Hart, 1997; Lubas et al., 1997; Gao et al., 2001). Notably, O-GlcNAcylation has been postulated to play a role in the regular operation of neurons. In contrast, its dysregulation is implicated in the development of neurological disorders. O-GlcNAcylation is believed to be an important mitigating factor of cellular stress responses. A previous report demonstrated prolonged survival times of rats undergoing experimentally induced hemorrhages after suppression of stress-induced hyperglycemia. The outcome after hemorrhage in 24-h food-deprived rats could be improved by pretreatment with a limited amount of oral carbohydrate solution 1 h prior to hemorrhagic stress (Nettelbladt et al., 1996). Initially, this protection was attributed to physical factors that maintained internal homeostasis or provided an adequate energy supply (Mizock, 2001). Due to the fact that UDP-GlcNAc is the final product, glucose flux through HBP is subsequently increased.

Inflammation is a significant factor contributing to secondary damage following ischemic stroke (Moskowitz et al., 2010). Post-ischemic stroke, resident microglia and astrocytes in the brain become activated. Circulating immune cells, including monocytes, neutrophils, and lymphocytes, are recruited to the site of injury, leading to the upregulation of various inflammatory mediators that collectively contribute to ischemic brain damage (Jin et al., 2010; Jin et al., 2013). Therefore, inhibiting the inflammatory response during cerebral ischemia can significantly improve the prospect of achieving better outcomes (Emsley and Hopkins, 2008). As a unique metabolic pathway, the O-GlcNAcylation is highly responsive to cellular stress (immune stress, oxidative and chemical stresses) (Zachara et al., 2004; Nagel and Ball, 2015; Martinez et al., 2017). GlcN or Thiamet-G -induced elevation of O-GlcNAc levels leads to a significant increase in neuroprotection in the ischemic brain by inhibiting the production of inflammatory cytokines and the activation of microglial cells (Hwang et al., 2010; He et al., 2017). The specific mechanism may involve the inhibition of NF-κB p65 signaling transduction. The similar effects of GlcN and Thiamet-G suggest that inhibiting inflammation may contribute to the neuroprotective mechanisms of O-GlcNAcylation. The nuclear factor kappaB (NF-κB) is primarily regulated through post-translational modifications, including phosphorylation, acetylation, and glycosylation. It modulates various cellular processes, including innate immunity, adaptive immunity, inflammation, cell apoptosis, cell survival, and differentiation. Five proteins comprise the nuclear factor NF-B transcription factor family: p65 (RelA), RelB, c-Rel, p105/p50 (NF-B1), and p100/p52 (NF-B2). Importantly, NF-κB is a typical pro-inflammatory signaling factor and serves as a key molecular bridge linking O-GlcNAcylation and inflammation. Increased tissue levels of O-GlcNAcylation modifications on proteins markedly attenuate the trauma-hemorrhage-induced elevation of the circulating inflammatory cytokines TNF-α and IL-6, while also inhibiting the activation of the NF-κB signaling pathway (Zou et al., 2009).

After activation, NF-κB is translocated to the nucleus, where it participates in the activation of NF-κB–dependent gene transcription. The increase of NF-κB levels in the nucleus is reflected by augmented NF-κB binding to consensus sequences (GGGATTTCCC). Electrophoretic mobility shift assay analysis demonstrates increased binding of nuclear proteins to the NF-κB consensus sequence in mesangial cells treated with high glucose, GlcN, or GFAT overexpression. This binding is associated with O-GlcNAcylation of p65, highlighting the relevance of O-GlcNAcylation in NF-κB signaling (James et al., 2002). Research has primarily concentrated on establishing the pro-inflammatory function of O-GlcNAcylation within the NF-κB signaling pathway. O-GlcNAcylation of p65 influences the interaction between NF-κB and its inhibitor IκB. IκBα undergoes phosphorylation at the S32 and S36 sites by IκB kinase upon stimulation by pro-inflammatory cytokines, lipopolysaccharide (LPS), or glucose, leading to inactivation of IκBα, followed by detachment and translocation of NFκBp65 from IκBα to the nucleus and binding to the NF-κB promoter/enhancer to initiate transcription (Sen and Baltimore, 1986a; Sen and Baltimore, 1986b; Oeckinghaus and Ghosh, 2009). This action was exploited in rat vascular smooth muscle, thereby enhancing NF-κB translocation to the nucleus and increasing VCAM-1 transcription under hyperglycemic conditions (Yang et al., 2008). NF-κBp65 O-GlcNAc modification, which is enhanced by GlcN or PUGNAc, inhibits the expression of inflammatory mediators induced by TNF-α in rat aortic smooth muscle cells (Xing et al., 2011). Thus, O-GlcNAcylation could also serve as a novel neuroprotective or anti-inflammatory tool that inhibits LPS-induced NF-κB activation by increasing the interaction between transcriptional corepressor mammalian Sin3A and OGT to reduce increased recruitment to NF-κB binding sites. O-GlcNAcylation also interferes with LPS-driven iNOS gene expression in macrophages (RAW264.7) (Hwang et al., 2013), and further inhibits LPS-induced upregulation of pro-inflammatory mediators, including TNF-α, lL-1β, IL-6, and COX-2 (Hwang et al., 2017). The O-GlcNAcylation modification activates the NF-κB subunit c-Rel (Ramakrishnan et al., 2013). In BV2 microglia cells, LPS stimulation increased c-Rel activation, O-GlcNAcylation of c-Rel, c-Rel binding to the NF-κB site of the iNOS promoter, and c-Rel interactions with OGT and p50/p105. These effects were inhibited by treatment with GlcN. The anti-inflammatory effects of GlcN were achieved by blocking prolonged activation of c-Rel and NF-κB transcription factors (Hwang et al., 2013).

Ischemia-induced stress initiates apoptosis subsequent to an ischemic stroke, with the primary site occurring in the ischemic semi-dark zone, resulting in neuronal cell death, microglial activation and initial tissue injury. Microglia undergo significant morphological and genetic changes when activated, in this process, microglia/macrophages divide into two distinct groups: the traditional M1 phenotype, responsible for generating pro-inflammatory cytokines that cause neuronal harm in ischemic brains, and the different M2 phenotype, which releases anti-inflammatory cytokines that inhibit immune reactions and aid in post-stroke recuperation. Nonetheless, the process of altering these phenotypes remains a matter of debate. He et al. (2017) concluded that in acute ischemic stroke, inhibition of OGA by Thiamet-G administration reduced infarct volume, ameliorated neurological deficits, and improved clinical outcomes. Mechanistically, Thiamet-G treatment modulated the expression of pro-inflammatory and anti-inflammatory cytokines by regulating microglia/macrophage polarization and inhibiting NF-κB p65 signaling. This implies that enhanced modification of O-GlcNAc acts as a crucial defense against harm caused by the immune system. Moderate elevation of O-GlcNAcylation has a neuroprotective effect by reducing infarct volume, motor deficits, and neurological deficits in mouse models of middle cerebral artery occlusion (MCAO) and ischemia/reperfusion (I/R). However, overly elevated levels of O-GlcNAcylation lead to more severe impairment of brain function. Gu et al. (2017) found that in mouse models of cerebral ischemia, treatment with a high concentration of Thiamet-G (resulting in O-GlcNAcylation levels increased more than 6-fold) worsened cerebral ischemic injury, caused changes in bleeding, and increased apoptosis. Conversely, treatment with GlcN or a low dose of Thiamet G (resulting in O-GlcNAcylation levels increased less than 3-fold) was neuroprotective. (Gu et al., 2017). They demonstrated that O-GlcNAcylation is both a response to cerebral ischemic injury and a novel regulation of cerebral ischemic-reperfusion injury. These findings suggest a novel therapeutic strategy for ischemic stroke through moderate pharmacological elevation of brain O-GlcNAcylation.

In a study of cerebral ischemia and hypoxia arising from MCAO, O-GlcNAcylation was found in the AKT signaling pathway. O-GlcNAcylation exhibited a negative correlation with phosphorylation levels at the Ser473 and Thr308 sites of AKT, and the increased level of O-GlcNAcylation and decreased level of phosphorylation led to decreased phosphorylation of the downstream molecule Bad, leading to increased expression of Bax and caspase3 proteins. Caspase3 proteins caused a cascade response to promote apoptosis, further verifying that heightened O-GlcNAcylation levels in the hippocampus coincided with neuronal apoptosis (Shi et al., 2015). Similar results were found in the MCAO model used to study excitotoxicity after cerebral ischemia, where O-GlcNAcylation of nNOS was significantly increased. Reducing O-GlcNAcylation of nNOS was neuroprotective by shielding neurons from apoptosis and decreasing neuronal death during glutamate stimulation by reducing the formation of the nNOS-post-synaptic density protein 95 complex, indicating a potential novel therapeutic approach for treating ischemic stroke (Chen et al., 2017).

Several studies have examined the kinetics and spatial distribution of O-GlcNAcylation and O-GlcNAc enzymes, suggesting a potential connection between fluctuating enzyme levels and brain development. Examination of O-GlcNAcylation levels in rat brains, from embryo to 2 years of age, revealed a post-birth decrease followed by stabilization (Rex-Mathes et al., 2001; Liu et al., 2012). The ability of the brain to activate survival pathways drastically decreased with age when challenged by ischemic stress in studies targeting post-translational modifications of proteins, including O-GlcNAcylation, which is altered in young and old mice undergoing surgically induced MCAO. Studies of clinical neuroprotection primarily involved young rodents, limiting their applicability to older stroke patients. The IRE1/XBP1/OGlcNAc axis emerges as a potential neuroprotective target in ischemic stroke, with Xbp1 deficiency exacerbating outcomes following transient and permanent MCAO. After a stroke, O-GlcNAcylation activation in young mouse stroke hemidesmosiderotic neurons is predominantly reliant on xbp1. In contrast, impaired O-GlcNAcylation in aging mouse brains, when compared to young mice, may contribute to slower recovery. These findings underscore the association between protein O-GlcNAcylation and age-related functional recovery following ischemic stroke, offering valuable insights for further research into O-GlcNAcylation during the initiation and progression of stroke (Liu et al., 2016; Jiang et al., 2017).

### 3.2 Role of O-GlcNAcylation in myocardial ischemia

The cardiovascular system is a susceptible target for I/R injury, which leads to irreversible cardiac dysfunction and injury, including various forms of cardiomyocyte death and coronary microvascular injury (Heusch, 2020).

O-GlcNAc glycosylation is essential for normal cardiac development (Dupas et al., 2021). Increasing evidence suggests that the O-GlcNAcylation plays an important role in myocardial ischemic injury. Upregulation of HBP flux is observed during episodes of acute myocardial I/R injury, which is cardioprotective (Dupas et al., 2021). Cardiomyocyte protein O-GlcNAc levels are elevated under ischemia, whereas reperfusion leads to a significant decrease in O-GlcNAc levels (Jensen et al., 2019). Increasingly, protein GlcNAc glycosylation in the heart is protective against I/R injury.

In neonatal rat cardiomyocytes, increasing HBP flux using GlcN or increasing O-GlcNAc levels using the OGA inhibitor PUGNAc enhances cell viability and attenuates cell necrosis and apoptosis after I/R injury and protein O-GlcNAc levels significantly correlate with cell survival during reperfusion (Champattanachai et al., 2007). Conversely, inhibition of HBP flux with azaserine (an inhibitor of GFAT) or OGT inhibitors such as Alloxan reversed these cardioprotective effects and prevented the increase in protein O-GlcNAc (Liu et al., 2007a).

In isolated perfused hearts, increasing cardiac HBP flux using GlcN or glutamine perfusion or increasing myocardial O-GlcNAc levels with the OGA inhibitor PUGNAc reduced I/R -induced myocardial injury (Liu et al., 2006; Liu et al., 2007a; Liu et al., 2007b). In addition, the protective effects associated with elevated myocardial O-GlcNAc levels and GlcN (among others) can be blocked by the action of the OGT inhibitor tetroxine or the GFAT rate-limiting enzyme inhibitor azaserine (Liu et al., 2007a). Ischemic preconditioning enhances O-GlcNAc levels *in vivo*, and the elevation of O-GlcNAc levels is sufficient to reduce infarct size after *in vivo* myocardial I/R injury (Jones et al., 2008). A prospective cohort study from UK Biobank demonstrated that habitual use of GlcN supplements to relieve osteoarthritis pain may be associated with a reduced risk of cardiovascular disease (Ma et al., 2019).

Collectively, these *in vitro*, *ex vivo* cardiac models, and *in vivo* investigations have emphasized the potential role of increased levels of the protein O-GlcNAc induced by enhanced HBP flux in the heart’s defense against cardiac I/R injury. Calcium overload, impaired mitochondrial function, endoplasmic reticulum stress, and oxidative stress are typical pathological features of I/R, and the mechanism of cardioprotection by O-GlcNAcylation is further explored mainly from these aspects:

(1) CaMKII plays a role in the damage caused by cardiac I/R injury and calcium overload leads to I/R injury by an increase in intracellular Ca^2+^ concentration during the I/R cascade and these Ca^2+^ oscillations results from repetitive Ca^2+^ uptake and release from the sarcoplasmic reticulum (Abdallah et al., 2011). In rat ventricular myocytes, overexpression of OGT results in elevated O-GlcNAc levels, mitigating cytoplasmic and mitochondrial calcium overload as well as oxidative stress induced by hypoxia and hydrogen peroxide. This effect was assessed through time-lapse fluorescence microscopy (Champattanachai et al., 2007; Ngoh et al., 2011). Inhibition of OGA expression by PUGNAc treatment was similarly attenuating; conversely, OGA overexpression reduced O-GlcNAc glycosylation and worsened hypoxia-induced Ca^2+^ overload. Increasing O-GlcNAcylation by treatment with GlcN blocks the excessive accumulation of cytoplasmic Ca^2+^ caused by angiotensin II (Nagy et al., 2006).
(2) Mitochondria play a vital role in supporting normal contractile function and cardiomyocyte metabolism in the heart, and I/R causes an increase in mitochondrial permeability and activation of the mitochondrial death pathway (Ngoh et al., 2011). Reperfusion-induced cardiomyocyte damage involves opening of the mitochondrial permeability transition pore, which may be the core of reperfusion injury (Perrelli et al., 2011). Numerous studies show that increasing cardiac O-GlcNAc levels significantly attenuates mitochondrial permeability transition pore and decreases cellular injury (Champattanachai et al., 2008; Hirose et al., 2011), although inhibiting OGT expression to decrease O-GlcNAc levels removes this protection. Elevated levels of apoptosis, accompanied by the release of pro-apoptotic factors, result from increased mitochondrial permeability, which also causes rupture and swelling of mitochondria (Halestrap, 2010; Ong et al., 2015). An increase in intracellular O-GlcNAc levels mitigates the loss of mitochondrial membrane potential induced by I/R, and OGT overexpression or treatment with PUGNAc to boost O-GlcNAc levels protects cardiomyocytes that were exposed to hypoxia-reperfusion from death (Ngoh et al., 2008; Ngoh et al., 2009a). Conversely, the suppression of OGT expression through pharmaceutical means, exacerbates the decline in mitochondrial membrane potential and leads to cell death.
(3) The endoplasmic reticulum (ER) is an important site for maintaining protein synthesis, folding, assembly, transportation, and participation in lipid metabolism. I/R-induced injury leads to impaired ER function, ER stress, accumulation of unfolded proteins, and apoptotic cell death (Wang et al., 2020). O-GlcNAcylation regulates I/R -induced ER stress and exerts a protective effect on cells. OGT overexpression or OGA inhibition increases O-GlcNAcylation levels, significantly reduces cardiomyocyte death, and ER stress (Ngoh et al., 2009b) and there is an elevated ER stress response in the c-cmOGT KO heart using a constitutive cardiomyocyte OGT-specific knockout mouse model (Watson et al., 2014). GlcN attenuates the upregulation of CHOP and GRP78 expression while reducing the proportion of apoptotic cells during hypoxia (Ngoh et al., 2009b). This observation implies that O-GlcNAc may safeguard cells against apoptosis induced by stress in the ER, potentially through the regulation of eIF2α phosphorylation. In HepG2 cells, enhancement of O-GlcNAc levels through Thiamet G treatment or OGT overexpression has demonstrated glycosylation of eIF2α at Ser 219, Thr 239, and Thr 241 sites, which impedes eIF2α phosphorylation and reduces CHOP activation, thereby reducing levels of apoptosis (Suh et al., 2014; Jang et al., 2015).
(4) Oxidative stress contributes to the development of numerous diseases, including myocardial injury. Low reactive oxygen species (ROS) concentrations are involved in intracellular signaling and regulation, thereby aiding in the preservation of cellular homeostasis (Yan et al., 2023), while reperfusion injury triggers an exponential surge in the generation of free radicals in isolated hearts (Maslov et al., 2023). Ischemic injury elicits an inflammatory response via the stimulation of neutrophil activation, the release of cytokines, and the activation of the complement system. In primary cardiomyocyte ischemia models, OGT overexpression or the increase of O-GlcNAcylation with OGA inhibitors attenuates calcium overload, oxidative stress, and mitochondrial damage caused by oxidative injury (Ngoh et al., 2011), inhibits apoptosis, and has a protective effect on cardiomyocytes, whereas OGA overexpression exacerbates hypoxia and oxidative damage. This implies that the inhibitory effect of moderately increased O-GlcNAcylation on ischemic cardiomyocytes might be associated with the suppression of calcium overload and accumulation of ROS in the heart. Hypoxic pre-acclimation reduces oxidative stress and protects against cardiac I/R injury and this protective effect is partly owing to the elevation of protein O-GlcNAc modification induced by inflammatory stimuli, which activates the pentose phosphate pathway and enhances redox homeostasis (Ou et al., 2021).

Alterations in O-GlcNAcylation levels have a direct impact on its protective effect. During myocardial ischemia, OGT expression increases and OGA expression decreases (Dassanayaka et al., 2020), which contributes to the elevation of O-GlcNAcylation to achieve protection. However, Ha et al. (2023) employed a mouse model with cardiomyocyte-specific dnOGA overexpression, resulting in increased O-GlcNAcylation protein levels. Persistently high levels of cardiac O-GlcNAc resulted in heart enlargement, heightened heart fibrosis, and impaired diastolic function. The phosphorylation of p38 induced by ischemia was considerably reduced in the presence of GlcN, implying that the cardioprotective effects of elevated protein O-GlcNAc levels may be achieved through the activation of the MAPK pathway (Fülöp et al., 2007).

Narayanan et al. (2023) found that O-GlcNAcylation was elevated in female mice during cardiac basal level and I/R, while OGT activity was sex-dependent during cardiac I/R and was enhanced in female mice compared to that in male mice. Their findings highlight the critical role of gender in evaluating essential regulatory processes that govern O-GlcNAc cycling (Narayanan et al., 2023). O-GlcNAcylation is excessively increased in certain pathological conditions, including diabetes mellitus (Jin et al., 2021b), which may aggravate myocardial injury.

In summary, O-GlcNAcylation regulation undergoes dynamic changes during myocardial ischemia, and a moderate increase in O-GlcNAcylation has a protective effect on the ischemic myocardium, while excessive O-GlcNAcylation leads to adverse outcomes. This provides important clues for the development of novel treatments for myocardial ischemia.

### 3.3 Role of O-GlcNAcylation in small intestinal ischemia

The occurrence of O-GlcNAcylation is common in ischemic conditions, particularly in the heart and brain, yet its involvement in ischemic intestinal disorders remains undocumented. Recent studies indicate that O-GlcNAcylation has a positive impact on I/R-induced intestinal injury (Cong et al., 2021). Furthermore, both *in vitro* and *in vivo* experiments demonstrate that the manipulation of O-GlcNAcylation levels through pharmacology has a significant impact on cell viability and tissue damage. In three-dimensional laboratory tests, enhancing O-GlcNAcylation boosted the resilience of IEC-6 cells to damage caused by hypoxia, whereas reducing O-GlcNAcylation lowered the survival rate of intestinal IEC-6 cells. Moreover, when the augmentation of O-GlcNAcylation was inhibited, the consequent protective effect against OGD/R-induced cellular injury was likewise nullified. *In vivo* experiments revealed that GlcN elevates intestinal protein O-GlcNAcylation and significantly alleviates the symptoms of intestinal injury in mice. O-GlcNAcylation also protects against small intestinal I/R injury by inhibiting apoptosis. Elevated GlcN-induced O-GlcNAcylation significantly reduces apoptosis as revealed by TUNEL staining and apoptosis protein immunoblotting (Cong et al., 2021). These findings indicate that O-GlcNAcylation has a broad protective role that may also be effective as a focus for future treatments for intestinal ischemic conditions.

### 3.4 Role of O-GlcNAcylation in renal ischemia

The kidney is highly susceptible to hypoxic injury due to its complex transport functions being performed within a narrow range of partial pressure of oxygen, which is particularly low in the medulla. To avoid hypoxic injury, adequate oxygen supply must be maintained to the kidney. Although renal cells can adapt to hypoxia to cope with changes in oxygen concentration using various molecularly mediated pathways, excessive hypoxia induced by ischemia can still damage the kidney.

Contrast-induced acute kidney injury is a complication among patients receiving intravascular contrast media, the passage of contrast media through the renal vascular bed leads to vasoconstriction, which results in decreased perfusion and ischemic injury to renal tubular cells (Bansal and Patel, 2020). Intraperitoneal administration of GlcN to increase O-GlcNAc, protects the kidney from iohexol (a low-osmolar contrast media)-induced injury and attenuates renal dysfunction, tubular injury, apoptosis and oxidative stress. Conversely, this protection was inhibited with alloxan (Hu et al., 2017). Hu et al. conducted another study that demonstrated how remote ischemic preconditioning-induced elevation of O-GlcNAc signaling improved contrast-induced acute kidney injury. Additionally, the use of alloxan, an O-GlcNAc transferase inhibitor, and azaserine, a glutamine fructose-6-phosphate amidotransferase inhibitor, neutralized the protective effects of RIPC against oxidative stress and apoptosis in renal tubular cells (Hu et al., 2018).

Sodium-dependent glucose transporters (SGLTs), SGLT1 and SGLT2 are responsible for glucose reabsorption in the kidney, and SGLT2 plays a major role. Hyperglycemia and hypoxia modulate the activity of SGLT in the kidney, and selective inhibition of SGLT2 reduces renal glucose reabsorption in hyperglycemic rats, though not in hypoglycemic or normoglycemic conditions (Nagata et al., 2013). In the rabbit model of ischemic acute kidney injury, the renal ischemia induced tubulointerstitial abnormalities and decreased SGLT expression in tubular brush-border, and these effects were reversed by GlcN (Suh et al., 2014). *In vitro* experiments have shown that hypoxia reduces O-GlcNAc protein levels and OGT expression, while increasing OGA expression, and GlcN reverses these conditions Hypoxia also reduced the expression of SGLTs, while pretreatment with GlcN and PUGNAc (an OGA inhibitor) restored the level of SGLTs (Suh et al., 2014). The potential connection between O-GlcNAc and SGLTs in enhancing resistance to hypoxic injury expands our comprehension of the role of O-GlcNAcylation in the mechanism of renal ischemic injury.

### 3.5 Role of O-GlcNAcylation in retinal ischemia

Retinal tissue is highly metabolically active and has a high susceptibility to ischemic lesions due to its inner blood supply originating from terminal branches of retinal blood vessels. This can be extremely harmful to vision as the visual neurons are located in the retinal tissue. Retinal I/R injury is a significant pathophysiological factor in various ischemic retinal diseases, including glaucoma, diabetic retinopathy (DR), and central retinal artery occlusion (Zhang et al., 2023). These diseases all result from inadequate blood perfusion in the retina, leading to local or extensive ischemia and hypoxia of the retina, which causes the death of numerous functional cells in the retina.

Acute glaucoma causes a rapid increase in intraocular pressure, which results in a range of retinal cellular injuries. These include poor retinal blood supply, I/R injury, metabolic and neurotrophic factor arrest, oxidative stress and immune response, apoptosis of retinal ganglion cells, and heightened vascular permeability (Szabo et al., 1991; Rabacchi et al., 1994; Hangai et al., 1995; Kerr et al., 2012; Huang et al., 2013). GlcN, as a major precursor of post-translational modification glycosylation, plays a regulatory role in the activation of the HBP, while GlcN protects retinal ganglion cells by regulating protein O-GlcNAc glycosylation and elevated O-GlcNAc levels exert anti-apoptotic, anti-inflammatory, and antioxidant effects to protect the retina. GlcN treatment had multiple protective effects on the retina in a rat retinal I/R model, including increasing the levels of anti-apoptotic Bcl-2 and O-GlcNAc modifications to regulate apoptosis, achieving anti-inflammatory effects by regulating NF-κB signaling-induced ICAM-1 expression, and increasing SOD-1 factors for antioxidant purposes (Chen et al., 2015). These results underscore GlcN as a potential retinal protective agent for the prevention of retinal I/R damage caused by acute glaucoma.

Diabetic retinopathy is a chronic complication of diabetes that commonly affects the neurovascular system of the retina, leading to severe vision problems, including blindness (Gurel and Sheibani, 2018). Analysis of the diabetic retinal proteome reveals that diabetes changes the global expression levels and O-glycosylation of metabolic and synaptic proteins in the retina (Starr et al., 2023). Kim et al. found that the expression of retinal O-GlcNAcylated proteins was significantly increased in a diabetic mouse model. Additionally, the p65 subunit of NF-κB was O-GlcNAcylated in the ganglion cell layer of the retina. This is involved in hyperglycemia-induced NF-κB activation and RGC death in DR (Kim et al., 2016; Kim et al., 2017). Diabetic retina is usually caused by diabetic macular edema, characterized by disruption of the blood-retinal barrier (Tan et al., 2017). Connexin43 is tightly linked to BRB function (Liu et al., 2021a). In diabetic rat retina and HRVEC cultured under high glucose conditions, O-GlcNAcylation negatively regulates connexin43 expression, which leads to the disruption of the blood-retinal barrier. However, inhibiting O-GlcNAcylation with alloxan significantly reduced hyperglycemia-induced vascular leakage by upregulating ZO-1 and occludin expression through the connexin43 pathway (Liu et al., 2023). Pericyte apoptosis is an early characteristic lesion of DR. Under hyperglycemic conditions, levels of O-GlcNAc modification are significantly elevated in retinal pericytes (Gurel and Sheibani, 2018). Gurel et al. (2014) discovered that post-translational O-GlcNAc modification of p53 and its elevated levels may cause selective early loss of pericytes during diabetes. Owing to the strong correlation between DR and O-GlcNAcylation, modulation of HBP flux and O-GlcNAc is expected to be a novel therapeutic strategy to prevent the onset and progression of DR.

### 3.6 O-GlcNAcylation in chronic metabolic diseases combined with ischemic diseases

Diabetes significantly contributes to the risk of heart disease and is linked to irregular control of O-GlcNAcylation (Ball et al., 2006). Wang et al. (2018) experimentally validated that diabetes-influenced hyperglycemia and hyperinsulinemia reduces plasma microRNA-24 levels in the hearts of diabetic mice more than in non-diabetic control mice during myocardial I/R, leading to lower survival rates and larger infarct sizes. OGT upregulation in high glucose cultured cardiomyocytes *in vitro* and OGT and O-GlcNAcylation in cardiac lysates *in vivo* were significantly reduced after overexpression of microRNA-24 by pharmacological or genetic engineering interventions, underwent coordinated downregulation, thus safeguarding the myocardium against ischemic heart disease (Wang et al., 2018). In addition, Liu et al. (2017) found that O-GlcNAc glycosylation of the important cardioprotective enzyme acetaldehyde dehydrogenase 2 (ALDH2) results in increased myocardial injury, cardiac dysfunction, and larger infarcts in rats with hyperglycemia following myocardial I/R. Pretreatment with the OGA inhibitor PUGNAc enhances the O-GlcNAcylation of ALDH2 and inhibits activity. In contrast, treatment with the GFAT inhibitor DON reduces the O-GlcNAcylation level of ALDH2 in cardiac myogenic cells of H9c2 rats. This resulted in a marked rise in ALDH2 activity and decrease in infarction area, apoptotic index, and degree of cardiac dysfunction induced by myocardial I/R combined with hyperglycemia *in vivo* (Liu et al., 2017). In human myocardium, the total protein O-GlcNAc modification was higher in diabetics than non-diabetics, and the modification was associated with left ventricular dysfunction. Prakoso et al. (2022) used cardiac-targeted recombinant adeno-associated viral vector-6 (rAAV6)-mediated gene delivery to investigate the effects of manipulating OGA and OGT on the diabetic cardiac phenotype. In non-diabetic mice, rAAV6-OGT impaired left ventricular diastolic function and induced maladaptive cardiac remodeling, including cardiac fibrosis, which recapitulates characteristics of diabetic cardiomyopathy. Conversely, rAAV6-OGA rescued left ventricular diastolic function and adverse cardiac remodeling in diabetic mice by protecting PI3K-Akt signaling in diabetic myocardium *in vivo* (Prakoso et al., 2022).

O-GlcNAcylation plays a crucial role in regulating cellular pathways that drive metabolism and responds to systemic metabolic alterations that are transmitted by hormonal signals to specific cells and tissues. O-GlcNAc signaling is closely linked to the regulation of several hormones linked to metabolic regulation, including gastric hunger hormone, insulin, and glucagon. The effects of insulin stimulation on the O-GlcNAc signaling pathway are particularly well studied since disruption of O-GlcNAc homeostasis has been implicated in the pathogenesis of insulin resistance (Hart et al., 2011; Sermikli et al., 2020). Insulin functions by attaching to insulin receptors on cell surfaces, particularly those of liver, muscle, and adipose tissues. Insulin acts as a suppressor of glycogen breakdown in both liver and muscle, promoting glycogen production and hindering gluconeogenesis within the liver. Insulin also maintains essential glucose homeostasis by stimulating glucose uptake by adipocyte and muscle cells. Inadequate insulin secretion is the reason for poor glycemic control, predisposing individuals to hyperglycemia (Yang et al., 2008). A number of studies have further confirmed the relationship between increased O-GlcNAcylation and insulin resistance (Dai et al., 2018; Shi et al., 2018; Sermikli et al., 2020). Marshall et al. (1991) connected heightened glucose flow via HBP to insulin resistance in peripheral tissues, a common occurrence in type 2 diabetes (Macauley et al., 2010; Ansari and Emerald, 2019). The alteration of O-GlcNAc is proposed as a potential pathway for insulin resistance and vascular issues associated with diabetes (Ansari and Emerald, 2019). Multiple lines of evidence suggest that O-GlcNAcylation is an influential factor in the development of diabetic nephropathy, and hyperglycemia-induced O-GlcNAcylation decreases eNOS and HSP72 expression by inducing inhibition of Akt phosphorylation sites and decreased kinase activity. Previous studies suggest that O-GlcNAcylation diminishes the renoprotective function of eNOS, thus elevating the likelihood of diabetic nephropathy and accelerating its advancement (Gellai et al., 2016). Mouse adipocytes that increased O-GlcNAcylation inhibited Tyr608 phosphorylation of IRS1, thereby downregulating Akt activity and promoting insulin resistance (Whelan et al., 2010).

Poor control of high body lipids exacerbates insulin resistance, which is also detrimental to recovery from cardiac I/R injury and risks inducing cardiac arrhythmias. O-GlcNAcylation is elevated in obese db/db mice and palmitate-induced insulin-resistant H9c2 cells. Additionally, increased O-GlcNAcylation by GlcN treatment attenuated insulin-induced cardioprotection, suggesting a mechanistic link between O-GlcNAcylation and insulin resistance. Conversely the suppression of O-GlcNAcylation by DON resensitised the insulin response, suggesting that O-GlcNAcylation as a potential therapeutic target for obesity-related diseases (Jin et al., 2021b). Moreover, it’s proposed that the diabetic hyperglycemic state leads to an increased flow through the HBP, resulting in elevated O-GlcNAcylation and ROS levels in H9c2 cardiomyoblasts under high glucose conditions compared to controls. This increase in O-GlcNAcylation and ROS levels is believed to enhance cardiomyocyte apoptosis, further damaging the heart over time (Rajamani and Essop, 2010). A trend toward increased O-GlcNAc levels was found in a study of isolated diabetic obese rats hearts compared to non-diabetic rats. Hypoglycemia increases the susceptibility of I/R damage to the heart in animals, regardless of their diabetic status. Hearts from diabetic animals were amenable to cardioprotection during hypoglycemia compared with hearts from animals without diabetes, presumably due to the endogenous protection that diabetic rats exhibited compared to normoglycemic rats, with a significant reduction in infarct size after I/R injury, though not during the hypoglycemic state (Pælestik et al., 2017). In conclusion, chronic elevated O-GlcNAcylation in diabetes is accompanied by diabetic complications and apparently participates in endogenous protection against I/R injury.

## 4 Summary and outlook

Ischemic diseases, marked by diminished blood flow leading to tissue-oxygen and nutrient deprivation, culminate in cellular demise and compromised organ function. Among the myriad post-translational modifications, O-GlcNAcylation, the addition of O-GlcNAc to serine or threonine residues, emerges as crucial in modulating protein functions under stress, thus playing an important role in ischemic pathology across various organs, including the brain, heart, kidneys, retina, and intestines. Diabetes complicates the role of O-GlcNAcylation in ischemic diseases, as it amplifies the risk of ischemic injury through vascular damage and impaired blood flow. Targeting O-GlcNAcylation modulation presents a promising therapeutic strategy to mitigate ischemic injury and specifically combat the increased risk and severity in diabetic patients. The protective capacity of O-GlcNAcylation in ischemia underscores its therapeutic potential, suggesting that controlling its levels could be a novel method to reduce ischemic damage and enhance patient prognosis across a spectrum of ischemic diseases.

Targeting O-GlcNAcylation through pharmacological intervention has significant therapeutic potential for ischemia. Numerous studies have demonstrated that increased O-GlcNAcylation under ischemic conditions can provide organ protection (Li and Yang, 2022). Common experimental drugs (as detailed in Table 1) include OGT inhibitors (including Alloxan, BZX, BADGP, OSMI 1–4), OGA inhibitors (including PUGNAc, NButGT, Thiamet G, and GlcNAcstatins), GFAT inhibitors (including DON and Azaserine), as well as other drugs (e.g., glutamine and GlcN). GlcN and Thiamet G effectively regulate O-GlcNAc levels and provide protection against I/R injury in rodent models (Hwang et al., 2010; He et al., 2017; Wang et al., 2021; Jhelum et al., 2022). GlcN is a commonly used nutritional supplement for joint cartilage to alleviate symptoms of osteoarthritis (Kolasinski et al., 2020). Regular GlcN supplementation is linked to a significant decrease in the risk of stroke (Mazzucchelli et al., 2022). However, the effectiveness of oral GlcN therapy remains controversial (Aghazadeh-Habashi and Jamali, 2011). In a recent longitudinal study, researchers found a significant association between GlcN use in osteoarthritis patients and increased risk of cardiovascular disease. However, statistically significant association was not observed between GlcN use and stroke (Yu et al., 2022). Our recent study demonstrated that O-GlcNAcylation exacerbates brain ischemic injury under hyperglycemic conditions, proposing a “double-edged sword” role of O-GlcNAcylation in ischemic diseases (Zhu et al., 2023). Although targeting O-GlcNAcylation for the treatment of ischemic diseases through pharmacological interventions holds considerable therapeutic potential, the lack of data on drug dosage and treatment duration remains a critical safety issue that needs to be addressed in clinical trials.

Site-specific studies of O-GlcNAc are helpful in understanding the functional role of protein O-GlcNAcylation. Initially, the O-GlcNAc aspect of identifying proteins was mainly undertaken using low-throughput methods (e.g., Edelman degradation and targeted mutagenesis). In recent years, mass spectrometry-based proteomics has become a sensitive and high-throughput tool for large-scale identification of O-GlcNAc proteins due to the development of enrichment and identification techniques (Ma et al., 2021b; Maynard and Chalkley, 2021). Ma et al. (2021a) created the O-GlcNAcAtlas (O-GlcNAc-specific database), which contains all experimentally identified O-GlcNAc loci and proteins from all species studied over the past 35 years (from 1984 to 31 Dec 2019). This has greatly facilitated the study of O-GlcNAcylation.

**FIGURE 2:**
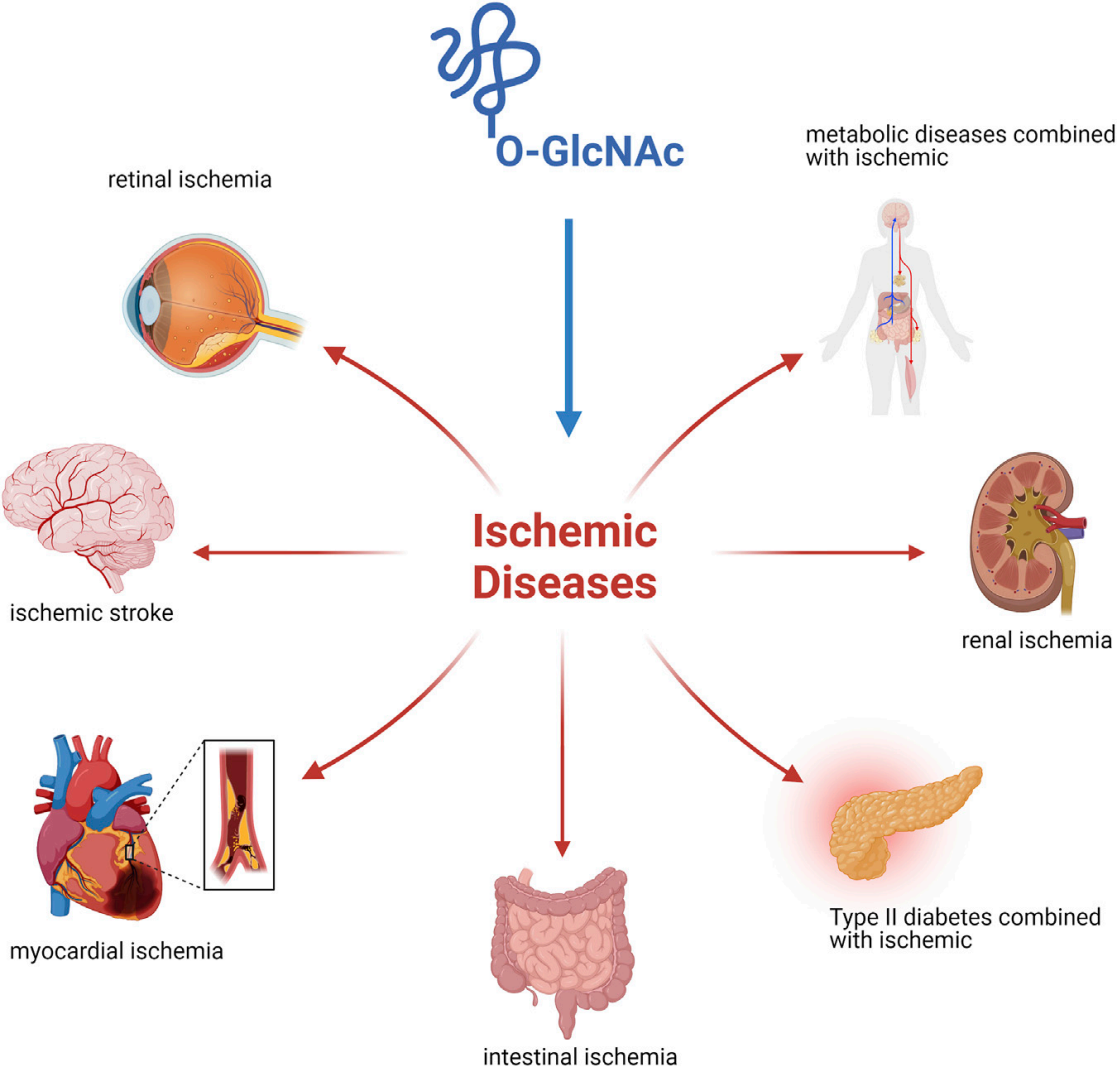
Ischemic disease injury is owing to tissue blood flow inadequate perfusion leading to localized hypoxic injury to tissues or cells and its regression is influenced by O-GlcNAc modification.

This review focused on O-GlcNAcylation as an emerging process after the occurrence of diseases combined with ischemia (Figure 2). The process of protein O-GlcNAcylation plays a crucial role in the progression of ischemic disease, serving as a vital connector between cellular activity and metabolism. There is growing evidence that the cycling enzymes of O-GlcNAc vary according to cellular conditions and stress at the time of onset, thereby causing fluctuations and alterations in O-GlcNAcylation, as observed in both *in vivo* and *in vitro* studies. Most experimental findings focus on alterations in O-GlcNAcylation levels instead of modifications to the underlying molecular processes. Finding ways to address the different elements that are associated with the pathogenesis of ischemic disease is the challenge we must address. Future studies in this field will yield more accurate insights into the regulation of O-GlcNAcylation and its specificity. We look forward to a thorough understanding of O-GlcNAcylation to establish a comprehensive network of metabolic and cellular activities that can restore health after damage to organismal systems. Furthermore, pharmacological interventions with O-GlcNAc cyclase inhibitors or studies using target knockout mice in an experimental setting may provide the optimal opportunity to develop therapeutic approaches to alleviate ischemic disease.

## Abbreviations

O-GlcNAc, oxygen-linked N-acetylglucosamine; GlcNAc, N-acetylglucosamine; UDP-GlcNAc, uracil-N-acetylglucosamine diphosphate; HBP, hexosamine biosynthesis pathway; GFAT, glutamine fructose-6-phosphate aminotransferase; OGT, O-GlcNAc transferase; OGA, O-GlcNAcase; DON, L-6-diazo-5-oxonorleucine; Azaserine, O-diazoacetyl-L-serine; TPR, tetrapeptide; BZX, 4-methoxyphenyl 6-acetyl-2-oxobenzo [d]oxazole-3(2H)-carboxylate; BADGP, Benzyl-2-acetamido-2-deoxy-α-d-galactopyranoside; OSMI 1, (R)-N-(furan-2-ylmethyl)-2-(2-methoxyphenyl)-2-(2-oxo-1,2-dihydroquinoline-6-sulfonamido)- N-(thiophen-2-ylmethyl)acetamide; PUGNAc, O-(2-acetamido-2-deoxy-D-gluco-pyranosylidene); NButGT, 1,2-dideoxy-2′-propyl-α-d-glucopyranoso-[2,1-D]-Δ2′-thiazoline; Thiamet G, 5H-Pyrano [3,2-d]thiazole-6,7-diol,2-(ethylamino)-3a,6,7,7a-tetrahydro-5-(hydroxymethyl) (3aR,5R,6S,7R,7aR); NF-κB, nuclear factor kappaB; LPS, lipopolysaccharide; MCAO, middle cerebral artery occlusion; I/R, ischemia-reperfusion; ER, endoplasmic reticulum; ROS, reactive oxygen species; SGLTs, Sodium-dependent glucose transporters; DR, diabetic retinopathy; ALDH2, acetaldehyde dehydrogenase; rAAV6, recombinant adeno-associated viral vector-6.
